# Acupuncture in Oncology: The Effectiveness of Acupuncture May Not Depend on Needle Retention Duration

**DOI:** 10.1177/1534735417734912

**Published:** 2017-11-02

**Authors:** Byeongsang Oh, Thomas Eade, Andrew Kneebone, George Hruby, Gillian Lamoury, Nick Pavlakis, Stephen Clarke, Chris Zaslawski, Isobel Marr, Daniel Costa, Michael Back

**Affiliations:** 1 Sydney Medical School, NSW, Australia; 2 University of Technology, Sydney, NSW, Australia; 3 University of Sydney Royal North Shore Hospital, NSW, Australia

**Keywords:** acupuncture, needle retention duration, dosage, quality of life, cancer

## Abstract

*Background.* Guidelines surrounding optimum needle retention duration in acupuncture have not been established, despite a growing evidence base for acupuncture over recent decades. This retrospective study explored the effect of varying acupuncture needle retention durations in cancer patients. *Method.* Patients received either 2 (n = 35), 10 (n = 53), or 20 minutes (n = 54) of acupuncture once a week for 6 weeks. Outcomes of anxiety and depression, stress, fatigue, and quality of life (QOL), with the Hospital Anxiety and Depression Scale, Perceived Stress Scale, Functional Assessment of Cancer Therapy–Fatigue, and European Organization for Research and Treatment of Cancer Quality of Life, were measured at baseline and at 6 weeks following the intervention. *Results.* The mean age of participants was 58 years (n = 152). The majority were female, diagnosed with breast cancer. Depression, stress, fatigue, and QOL were significantly improved in all 3 groups at 6 weeks postintervention. No significant differences in all outcomes were found between the 3 groups (≤2 vs 10 minutes vs 20 minutes). There were no differences with the satisfaction of the acupuncture services and perceived efficacy of acupuncture among the 3 groups. More than 95% of participants indicated that they would recommend acupuncture to other cancer patients, friends, and their family members. *Conclusion.* The efficacy of acupuncture may not only depend on needle retention duration, but may also be associated with multiple factors. Considering the limitations of this study design, robust randomized controlled studies are warranted to confirm the findings.

## Introduction

Cancer is a new epidemic in modern society.^1,2^ Whereas advances in biomedicine have increased cancer patients’ survival over recent decades, some individuals receiving cancer treatment experience significant adverse effects during treatment. In addition to the adverse effects caused by cancer treatment, many people diagnosed with cancer suffer from anxiety, depression, and fear of recurrence.^3,4^ Thus, a high proportion of patients use complementary and alternative medicine (CAM), including acupuncture to manage their physical and psychological symptoms.^5,6^

Acupuncture has become a popular therapy to manage adverse effects during cancer treatment in recent years.^7,8^ Emerging evidence from clinical trials has shown that acupuncture can alleviate symptoms of adverse effects during and post–cancer treatment. Several randomized controlled trials (RCTs) have evaluated acupuncture in the management of adverse effects and reported beneficial impacts on depression,^9^ chemotherapy-induced nausea and vomiting,^10^ chemotherapy-related neutropenia,^11^ and chemotherapy-related hot flushes.^12,13^ Studies have also demonstrated that acupuncture can reduce fatigue,^14^ joint pain and stiffness,^15,16^ and radiation-induced xerostomia^11^ and enhance overall well-being.^17^

Although several previous studies have demonstrated the value of acupuncture in cancer care, few studies have examined the relationship between dosage (needle retention duration, frequency, and total acupuncture dose) and clinical outcomes.^18^ Two classical textbooks on acupuncture described needle retention durations as brief as 3 exhalations^19^ and 6 exhalations^20^ for certain acupuncture points. Several studies conducted for pain management have demonstrated that the acupuncture intervention induces an analgesic effect irrespective of dose delivered.^21^ In contrast, 1 observational study reported that the pain threshold increased with persistent needle stimulation and induced an analgesic effect between 30 and 40 minutes.^22^ With the dearth of standard acupuncture needle retention duration guidelines, trials have often used varying lengths (median of 30 minutes; range from 15 to 60 minutes).^23^

To date, acupuncture needle retention duration has not received great attention in clinical practice because of the philosophical basis of acupuncture. Generally, in acupuncture, as with other CAM therapies, a greater weight is placed on personalized treatment methods based on individual symptoms rather than a unified standard treatment. Furthermore, standardized, evidence-based acupuncture practice guidelines in relation to needle retention duration have yet to be established. In recognition of this gap, this study evaluated the effects of needle retention duration on clinical outcomes during systemic and radiation cancer treatment. We hypothesized that acupuncture needle retention duration is associated with the effect of acupuncture on clinical outcomes (anxiety, depression, stress, fatigue, and quality of life [QOL]) of patients during systemic and radiation cancer treatment.

## Patients and Method

### Patients and Setting

The medical records and the data collected to evaluate acupuncture services in the Northern Sydney Cancer Centre (NSCC) of the Royal North Shore Hospital (RNSH) were reviewed. Six sessions of acupuncture treatment were offered to patients free of charge at NSCC between June 2014 and May 2016. Eligibility criteria to receive acupuncture service were diagnosis of cancer (at any stage), age 18 years or older, completion of or current treatment with surgery, chemotherapy and/or targeted therapies and/or radiotherapy or hormone therapy, and the ability to complete the questionnaire. Exclusion criteria included previous use of acupuncture during cancer treatment in the past 6 weeks, severe coagulopathy or bleeding disorder or dermatological disease within the acupuncture area, active infection and needle phobia rendering patient unable to receive acupuncture, and inability to understand the nature of the study sufficiently to allow completion of all study assessments.

### Procedure

Patients were given information about the availability of acupuncture services by oncologists, care coordinators, nurses, and receptionists during their hospital visit. If patients were interested in receiving acupuncture, they were advised to make a booking for acupuncture treatment at reception.

Patients received a total of 6 sessions of acupuncture once a week over 6 weeks. The acupuncturist provided different acupuncture needle retention durations in the 3 groups (needle retention duration for first, second, and third groups were 20 minutes after 10 minutes of consultation, 10 minutes plus 10 minutes bedrest after 10 minutes of consultation, 2 minutes plus 18 minutes of bedrest after 10 minutes of consultation, respectively); the total acupuncture intervention period was identical in all 3 groups. During the period of August 2014 to September 2015, patients were allocated to either group 1 or group 2 based on their first acupuncture appointment; 8 patients were booked for each day. In the patient’s booking list, odd numbers (1, 3, 5, and 7) and even numbers (2, 4, 6, and 8) were assigned to groups 1 and 2, respectively. Patients who received acupuncture during the period of October 2015 to March 2016 were allocated to group 3. Patients were given the questionnaire by receptionists prior to the acupuncture consultation and 6 weeks after completion of acupuncture treatment.

The preintervention questionnaire consisted of demographic information (gender, age, ethnicity, education, income, occupation, cancer diagnosis, extent of disease, and medical treatment received as well as major reasons that patients were considering having acupuncture) and anxiety, depression, stress, fatigue, and quality-of-life questionnaires. The postintervention questionnaire asked about the change of outcomes (anxiety, depression, stress, fatigue, and QOL) and included additional information to evaluate the acupuncture services (perceived efficacy and adverse effects of acupuncture, and satisfaction with acupuncture services). Ethics approval was obtained from the Human Research Ethics Committee of the RNSH.

### Study Design and Outcomes

This was a retrospective exploratory pilot study. Data collected to evaluate acupuncture services from June 2014 to May 2016 were analyzed. The effects of acupuncture on anxiety and depression, stress, fatigue, and QOL were measured with validated reported outcome instrument tools: the Hospital Anxiety and Depression Scale (HADS), Perceived Stress Scale (PSS), Functional Assessment of Cancer Therapy Fatigue (FACT-Fatigue), and the European Organization for Research and Treatment of Cancer Quality of Life (EORTC QLQ-C30). In addition, single items of EORTC QLQ-C30 were applied to assess individual symptoms. The HADS contains the Anxiety and Depression subscales.^24^ HADS was found to perform well in assessing the symptom severity of anxiety disorders and depression in both somatic, psychiatric, and primary care patients and in the general population. The PSS is commonly used in health and behavioral health contexts for examining patient-reported stress experiences and psychoneurological responses.^25^ Cancer-related fatigue is generally assessed by the FACT-Fatigue.^26^ The EORTC QLQ-C30 is a questionnaire developed by the European Organization for Research and Treatment of Cancer to assess the QOL of cancer patients. This 30-item instrument has 5 functional scales (physical, role, cognitive, emotional, and social), 3 symptom scales (fatigue, pain, and nausea and vomiting), a global health and QOL scale and a number of single items assessing additional symptoms commonly reported by cancer patients (dyspnea, loss of appetite, insomnia, constipation, and diarrhea), and perceived financial impact of the disease. Questions 1 to 28 have 4-point response scales—namely, “Not at all,” “A little,” “Quite a bit,” and “Very much.” Questions 29 and 30 record global health status on a 7-point response scale (EORTC QOL-C30 Scoring Manual, 2001).^27^

### Intervention

Patients received individualized acupuncture treatments based on their main complaint and ongoing cancer treatment. They were allowed to take their usual medication prescribed by their oncologists and general practitioner as needed and to include other CAM while they received acupuncture treatment. During the consultation, the acupuncture practitioner evaluated patients’ clinical symptoms. Participants in all 3 groups received bilateral perpendicular insertion of sterile disposable needles (Seirin J-type made in Japan, gauge and size 0.12 × 15 mm^2^) at various acupuncture points. The needling technique included twirling, thrusting, and lifting. The designation of acupuncture points adhered to the first edition of the Standard Acupuncture Nomenclature.^28^ Acupuncture was administered by a qualified acupuncture practitioner (BO), who had more than 15 years of acupuncture experience, was registered by the Chinese Medicine Board of Australia (CMBA), and had received accreditation from the RNSH and oncology acupuncture training.

### Data Analysis

Descriptive statistics were used to characterize the demographic profile of patients among 3 groups. The outcomes measured by the HADS, PSS, FACT-F, and EORTC QLQ-C30 at 2 time points (baseline and week 6) were analyzed. Because the outcomes generally had skewed distributions, we used nonparametric tests to analyze change over time and difference between groups. The Wilcoxon test was used to compare preintervention with postintervention data on the combined sample. For group comparisons, we calculated pre-post intervention change scores and first conducted Kruskal-Wallis tests to determine whether there were overall group differences, followed by Mann-Whitney *U*-tests on each pair of groups (ie, 2 vs 10 minutes, 2 vs 20 minutes, 10 vs 20 minutes). We used this strategy rather than a single Kruskal-Wallis test to examine specific group comparisons rather than overall differences between groups. All analyses were conducted using SPSS v22.

## Results

### Demographic Characteristics of Participants

A total of 142 patients received either 2 (n = 35), 10 (n = 53), or 20 minutes (n = 54) of acupuncture treatment once a week for 6 weeks. The demographic characteristics of the participants are presented in Table 1. A χ^2^ analysis shows that there were no significant differences in age, gender, ethnicity, education level, income, occupation, cancer diagnosis, and duration of cancer diagnosis among the 3 groups.

**Table 1.** Patient Characteristics.^a^

Variables	Acupuncture Dosage Level (Treatment Time)			*P* Value
	≤2 Minutes (n = 35), Frequency (%)	10 Minutes (n = 53), Frequency (%)	20 Minutes (n = 54), Frequency (%)	
Age, years (mean ± SD)^b^	59.2 ± 10.6	57.2 ± 9.7	57.2 ± 11.9	.699
Gender				
Male	7 (20)	12 (23)	10 (19)	.867
Female	28 (80)	41 (77)	44 (81)	
Ethnicity				
Caucasian	29 (83)	37 (79)	45 (88)	.031
Other	6 (17)	10 (21)	6 (12)	
Educational level				
Primary	0 (0)	0 (0)	2 (4)	.287
Secondary	7 (20)	8 (16)	13 (25)	
Tertiary	28 (80)	43 (84)	37 (71)	
Income level ($AUD)				
<30 000	10 (32)	14 (30)	12 (23)	.888
<30 000 To <60 000	5 (16)	6 (13)	9 (17)	
<60 000 To <100 000	9 (29)	15 (33)	14 (27)	
>100 000	7 (23)	11 (24)	17 (33)	
Occupation				
Manager/Professional	13 (39)	23 (47)	23 (45)	.181
Tradesperson	0 (0)	4 (8)	1 (2)	
Salesperson/Clerk	2 (6)	3 (6)	4 (8)	
Home duties	2 (6)	6 (12)	6 (12)	
Retired	12 (36)	9 (18)	17 (33)	
Unemployed	4 (12)	4 (8)	0 (0)	
Primary cancer diagnosis				
Lung	2 (6)	3 (6)	1 (2)	.897
Prostate	4 (11)	3 (6)	5 (10)	
Breast	19 (54)	31 (62)	34 (65)	
Brain	1 (3)	1 (2)	0 (0)	
Ovary	2 (6)	2 (4)	0 (0)	
Colorectal	1 (3)	3 (6)	4 (8)	
Other	6 (17)	7 (14)	8 (15)	
Extent of disease				
Localized	25 (78)	35 (73)	41 (80)	.653
Metastasis	7 (22)	13 (27)	10 (20)	
Time since diagnosis				
<12 Months	21 (60)	27 (54)	38 (73)	.172
>12 Months to 5 years	13 (37)	17 (34)	11 (21)	
>5 Years	1 (3)	6 (12)	3 (6)	
Treatment received^c^				
Surgery	28 (78)	40 (76)	42 (78)	.951
Chemotherapy	17 (47)	32 (60)	32 (60)	.416
Radiotherapy	29 (81)	44 (83)	43 (80)	.9
Hormonal therapy	8 (22)	20 (38)	13 (24)	.181
Palliative care	0 (0)	1 (2)	1 (2)	.711
Other	3 (8)	2 (4)	2 (4)	.543

a *P* values (χ^2^ test) are for comparison among groups allocated for acupuncture treatments (≤2 vs 10 vs 20 minutes).

b One-way ANOVA test was conducted for comparison of age.

c Participants received more than 1 anticancer treatment.

### Main Reasons for Considering Acupuncture Use

The most common reasons that participants gave for considering acupuncture use were fatigue, pain relief, difficulty sleeping, and neuropathy symptoms (Table 2).

**Table 2.** Main Reasons for Considering Acupuncture.^a^

Variables	Acupuncture Dosage Level (Treatment Time)			*P* Value
	<2 Minutes (n = 35), Frequency (%)	10 Minutes (n = 53), Frequency (%)	20 Minutes (n = 54), Frequency (%)	
Fatigue	16 (44.4)	27 (50.9)	35 (64.8)	.223
Lack of energy	16 (44.4)	18 (34)	27 (50)	.197
Improve immune function	6 (16.7)	12 (22.6)	17 (31.5)	.396
Pain	11 (30.6)	26 (49.1)	25 (46.3)	.112
Difficulty sleeping	14 (38.9)	18 (34)	28 (51.9)	.279
Anxiety	5 (13.9)	10 (18.9)	17 (31.5)	.050
Neuropathy (tingling in hands and feet etc)	13 (36.1)	14 (26.4)	18 (33.3)	.584
Depressed mood	5 (13.9)	10 (18.9)	14 (25.9)	.179
Improve quality of life	3 (8.3)	11 (20.8)	12 (22.2)	.303
Headaches	2 (5.6)	4 (7.5)	9 (16.7)	.300
Nausea	2 (5.6)	8 (15.1)	11 (20.4)	.246
Lack of enjoyment of life	1 (2.8)	6 (11.3)	14 (25.9)	.035^b^
Constipation	4 (11.1)	8 (15.1)	10 (18.5)	.739
Diarrhea	1 (2.8)	6 (11.3)	11 (20.4)	.093
Lack of appetite	0 (0)	5 (9.4)	9 (16.7)	.077
Hot flush	10 (27.8)	9 (17)	7 (13)	.352
Other reasons	5 (13.9)	8 (15.1)	4 (7.4)	.295

a *P* values (χ^2^ test) are for comparison among groups allocated for acupuncture treatments (≤2 vs 10 vs 20 minutes).

b *P* < 0.05.

### Outcome Measurement

There were significant improvements from preintervention to postintervention for depression (*P* < .001), stress (*P* < .001), fatigue (*P* < .001), and QOL (*P* < .001) 6 weeks postintervention but no significant effects on anxiety (*P* = .072). There were no overall significant differences in anxiety, depression, stress, fatigue, and QOL between the 3 groups postintervention (Table 3). Subgroup analyses were conducted to compare differences between each group (2 vs 10 minutes, 2 vs 20 minutes, and 10 vs 20 minutes). In a subgroup analysis comparing the 2- and 10-minute groups, the latter intervention group showed a significant reduction in fatigue (*P* < .05) and improvement in role function (*P* < .05) compared with the 2-minute intervention group, whereas there were no differences in other outcomes. In a comparison between 2 and 20 minutes, anxiety (*P* < .05), fatigue (*P* < .05), role function (*P* = .01), and QOL (*P* < .05) were significantly improved in the 20-minute intervention group compared with the 2-minute intervention group. However, there were no differences in any outcomes between the 10- and 20-minute intervention groups.

**Table 3.** Comparison Between 3 Treatment Time Conditions, Showing Change Scores for Patient Outcomes.

Variables	Acupuncture Treatment Time			Mann-Whitney *U*-Tests Comparing Each Pair of Groups						Preintervention to Postintervention on the Combined Sample	
	<2 Minutes	10 Minutes	20 Minutes	<2 vs 10 Minutes		<2 vs 20 Minutes		10 vs 20 Minutes			
	Median Change			*U* ^a^	*P* Value	*U*	*P* Value	*U*	*P* Value	W^b^	*P* Value
Stress measured by PSS											
Stress	1	2	2	251.5	.39	259	.30	481.5	.69	4.22	<.01^c^
Anxiety and depression measured by HADS											
Anxiety	0	0.5	1	230.5	.10	223.5	.03^c^	407.5	.22	1.80	.07
Depression	1	1.5	1	242.5	.16	307.5	.48	413.5	.25	4.54	<.01^c^
Fatigue measured by FACT-F^c^											
Fatigue	0.5	5	5	212	.03^c^	187	.02^c^	459.5	.48	5.10	<.01^c^
QOL measured by EORTC QLQ-C30^c^											
Physical function	0	6.67	6.67	298	.81	255.5	.36	412	.43	4.18	<.01^c^
Role function	8.33	0	0	205.5	.04^c^	169	.01^c^	434	.64	2.12	<.03^c^
Cognitive function	0	16.67	0	285.5	.34	324.5	.82	424.5	.22	3.58	<.01^c^
Emotional function	0	0	8.33	295.5	.45	301.5	.52	506	.94	3.03	<.01^c^
Social function	0	16.67	0	288	.36	295	.42	499	.86	3.93	<.01^c^
Global QOL	0	8.33	8.33	281.5	.32	229	.04^c^	440	.33	3.90	<.01^c^
Symptoms measured by EORTC QLQ-C30^c^											
Pain	0	0	16.67	307.5	.59	249	.10	424.5	.22	3.76	<.01c
Nausea/vomiting	0	0	0	246	.16	269.5	.44	404.5	.31	2.06	<.04^c^
Dyspnea	0	0	0	267	.59	266	.43	418	.77	1.94	.05
Sleeping	0	0	0	293.5	.72	284	.73	414.5	.44	3.16	<.01^c^
Appetite	0	0	0	291	.65	278	.56	403	.26	1.04	.30
Constipation	0	0	0	254	.23	227	.08	452	.84	1.71	.09
Diarrhea	0	0	0	298.5	.40	282	.18	492	.72	2.20	.03^c^

Abbreviations: PSS, Perceived Stress Scale; HADS, Hospital Anxiety and Depression Scale; FACT-F, Functional Assessment of Cancer Therapy–Fatigue; QOL, quality of life; EORTC QLQ-C30, European Organization for Research and Treatment of Cancer Quality of Life.

a U: Mann-Whitney U-tests were performed to compare between the groups.

b W: Wilcoxon test was used to compare preintervention to postintervention on the combined sample.

c *P* < 0.05.

### Evaluation of Acupuncture Services

Additional information was collected from participants to audit the acupuncture services 6 weeks post–acupuncture intervention (Table 4). There were no group differences in satisfaction with acupuncture services, perceived efficacy of acupuncture, side effects, and willingness to continue with acupuncture or to recommend the acupuncture service to other cancer patients. More than 95% of participants indicated that they were satisfied with the service and would recommend acupuncture to other cancer patients, friends, and family members. The responses to the query on how they perceived the effectiveness of acupuncture were as follows: effective, 70%; not effective, 29%; and difficult to judge, 1%.

**Table 4.** Evaluation of Acupuncture Services.^a^

Variables	Acupuncture Dosage Level (Treatment Time)			*P* Value
	<2 Minutes (n = 35) Frequency (%)	10 Minutes (n = 53) Frequency (%)	20 Minutes (n = 54) Frequency (%)	
Was acupuncture effective?				
Yes	12 (60)	22 (66.7)	27 (79.4)	.246
No	7 (35)	11 (33.3)	7 (20.6)	
Difficult to judge	1 (5)	0 (0)	0 (0)	
Experienced side effect?				
Yes	1 (5)	4 (12.1)	2 (5.9)	.067
No	14 (70)	26 (78.8)	32 (94.1)	
Difficult to judge	4 (20)	3 (9.1)	0 (0)	
Satisfied with acupuncture services?				
Highly satisfied	14 (70)	25 (75.8)	27 (79.4)	.630
Satisfied	5 (25)	7 (21.2)	4 (11.8)	
Partially satisfied	1 (5)	1 (3)	3 (8.8)	
Not satisfied	0 (0)	0 (0)	0 (0)	
Carry on acupuncture				
Definitely	11 (55)	14 (42.4)	14 (41.2)	.749
Probably	4 (20)	13 (39.4)	11 (32.4)	
Not sure	5 (25)	5 (15.2)	8 (23.5)	
Probably not	0 (0)	1 (3)	1 (2.9)	
Definitely not	0 (0)	0 (0)	0 (0)	
Recommend our acupuncture to other cancer patients				
Definitely	17 (89.5)	26 (78.8)	30 (88.2)	.322
Probably	1 (5.3)	6 (18.2)	4 (11.8)	
Not sure	1 (5.3)	0 (0)	0 (0)	
Probably not	0 (0)	1 (3)	0 (0)	
Definitely not	0 (0)	0 (0)	0 (0)	
Recommend acupuncture to your friend and your family members?				
Definitely	13 (68.4)	25 (75.8)	30 (88.2)	.286
Probably	5 (26.3)	7 (21.2)	4 (11.8)	
Not sure	1 (5.3)	0 (0)	0 (0)	
Probably not	0 (0)	1 (3)	0 (0)	
Definitely not	0 (0)	0 (0)	0 (0)	

a *P* values (χ^2^ test) are for comparison among groups allocated for acupuncture treatments (≤2 vs 10 vs 20 minutes).

## Discussion

The importance of needle retention duration on clinical outcomes was described in early acupuncture classical textbooks “*The Systematic Classic of Acupuncture and Moxibustion*”^19^ published during the Jin dynasty (265-420 ce) and “*The Great Compendium of Acupuncture and Moxibustion*”^20^ published during the Ming dynasty in 1601. The authors recommended retention times as brief as 3 exhalations^19^ and 6 exhalations^20^ for certain acupuncture points to treat particular medical conditions and manage symptoms. Despite multiple studies demonstrating the potential benefits of acupuncture over the past 2 decades, no trials in Western countries have investigated the association between acupuncture needle dosage (needle retention duration, frequency, and total acupuncture dose) and clinical outcomes in cancer populations. The current retrospective study examined the effect of acupuncture needle retention duration on clinical outcomes (anxiety, depression, stress, fatigue, and QOL). The most notable feature of the current study is that to our knowledge this is the first exploratory study examining the relationship between acupuncture needle retention duration and clinical outcomes in cancer patients. Moreover, the results of the study provide valuable information for use in designing future RCTs with acupuncture.

The study found that outcomes were significantly improved in all 3 groups 6 weeks postintervention. Most outcome measures showed no significant differences between the 3 groups (≤2 vs 10 vs 20 minutes); however, 2 minutes was inferior in terms of fatigue and role function when compared with 10 minutes and, additionally, anxiety and QOL when compared with 20 minutes. This, combined with the lack of significant differences between 10 and 20 minutes, suggests that the effects of acupuncture on clinical outcomes in cancer patients may not depend on the needle retention duration once a certain threshold is reached. The findings of the present study are in agreement with Hansen’s study,^29^ which examined the effects of acupuncture (5 vs 20 minutes) in patients with chronic neck and/or shoulder pain. Participants received a 10-week course of acupuncture and found no differences in pain improvement between the groups at 10-week and 6-month follow-ups. The current results are also comparable to recent acupuncture guidelines and the results of surveys. A resource specifically designed for providing treatment to patients with cancer recommended 15 minutes of needle retention duration.^30^ A recent survey assessed the views from patients treated with acupuncture in 10 minutes of appointments by an individual general practitioner in a National Health Service general practice and showed beneficial effects on pain and QOL.^31^ Conversely, Lundeberg et al^32^ used acupuncture treatment for patients with chronic head and neck pain and suggested that pain improved more in those receiving 30 to 60 minutes of acupuncture than 1 to 5 minutes. Issues with this finding include study design weaknesses such as the randomization of multiple groups (manual acupuncture, superficial acupuncture, and low- and high-frequency electroacupuncture) during a relatively short intervention period testing several outcomes.

The present findings may have applications in optimizing acupuncture provision in a resource-sensitive public health care system. For example, greater use of acupuncture services could be achieved with 15-minute appointments, in contrast to the usual 30 to 60 minutes. However, the results need to be interpreted with caution because they do not rule out the possibility that longer needle retention duration may be more effective in certain medical conditions. Although this study found that there are no major differences in clinical outcomes in relation to needle retention duration, the total acupuncture intervention duration (30 minutes per week for 6 weeks) in the 3 groups were identical. The first, second, and third group received, respectively, 10 minutes consultation and 20 minutes acupuncture treatment, 10 minutes consultation, 10 minutes treatment and additional 10 minutes bedrest, and 10 minutes consultation, 2 minutes treatment, and 18 minutes bedrest. There is a possibility that the confounding factors (bedrest and doctor-patient relationship; lifestyle changes, including diet; and use of other complementary therapies) may have partially contributed to the positive impact on clinical outcomes in addition to the acupuncture needle retention duration. Furthermore, considering these confounding factors, it is difficult to judge the true effect of acupuncture on the outcomes. Another limitation of the current study is its retrospective study design. Unlike RCTs, the current study did not control for potential confounding factors and bias. For example, participants were not a homogeneous group, but rather included those with diagnoses in multiple cancers and different stages of cancer and different treatments (chemotherapy, radiation therapy, and hormone therapy). Nonetheless, taking into account the discussion by Patsopoulos^33^ on the advantages and disadvantages of retrospective study, an advantage of the current study is that it represents an evaluation of an intervention in real-life routine practice conditions that can be generalized and applied to similar settings. In addition, to compensate for the weaknesses of a retrospective study, the researchers collected multiple data points (demographic profile and reasons for treatment), clinical outcomes (anxiety, depression, stress, fatigue, and QOL), and perceived benefits of acupuncture and satisfaction with acupuncture services to minimize the effects of bias and confounding factors in the study. Another weakness of this study was that it involved a short-term intervention. Patients were eligible to receive 6 sessions of acupuncture once per week, compared with other acupuncture trials, which include 12 weeks of treatment and long-term follow-up.^12,16^

Despite these limitations, the central study outcome of needle retention duration and efficacy was masked to participants. The researcher informed all participants that the study was designed to evaluate the effects of acupuncture on management of adverse cancer treatment effects and to collect feedback about the acupuncture service. To test the effect of masking in the trial, data relating to participants’ perception of and satisfaction with acupuncture services were collected at the end of the intervention in addition to multiple clinical outcomes. Data analysis showed that there was a balance in participants’ perception and satisfaction with acupuncture services among the 3 groups, and this demonstrated reliability of the masking effect. This suggests that the impact of confounding factors and bias on clinical outcomes in this study might not be excessive. Nevertheless, further robust RCTs with long-term follow-up are required prior to making an evidence-based recommendation about appropriate needle retention duration and/or dosage.

In conclusion, acupuncture has the potential to improve the QOL of cancer patients. Efficacy or benefits of acupuncture may not only depend on needle retention duration, but may also result from multiple factors associated with clinical outcomes. Although the current study does not provide sufficient evidence to establish acupuncture practice guidelines for needle retention duration, the findings provide critical information for the use of acupuncture in cancer care. Robust RCTs are warranted to confirm the findings.
